# Selective Prefrontal Disinhibition in a Roving Auditory Oddball Paradigm Under *N*-Methyl-D-Aspartate Receptor Blockade

**DOI:** 10.1016/j.bpsc.2018.07.003

**Published:** 2019-02

**Authors:** Richard E. Rosch, Ryszard Auksztulewicz, Pui Duen Leung, Karl J. Friston, Torsten Baldeweg

**Affiliations:** a Wellcome Trust Centre for Neuroimaging, University College London, London, United Kingdom; b Developmental Neurosciences Programme, UCL Great Ormond Street Institute of Child Health, University College London, London, United Kingdom; c Department of Biomedical Sciences, City University of Hong Long, Hong Kong

**Keywords:** Dynamic causal modeling, EEG, ERP, Event-related potential, Ketamine, Mismatch negativity, NMDA receptor

## Abstract

**Background:**

Disturbances in *N*-methyl-D-aspartate receptors (NMDARs)—as implicated in patients with schizophrenia—can cause regionally specific electrophysiological effects. Both animal models of NMDAR blockade and clinical studies in patients with schizophrenia have suggested that behavioral phenotypes are associated with reduction in inhibition within the frontal cortex.

**Methods:**

Here we investigate event-related potentials to a roving auditory oddball paradigm under ketamine in healthy human volunteers (*N=* 18; double-blind, placebo-controlled, crossover design). Using recent advances in Bayesian modeling of group effects in dynamic causal modeling, we fit biophysically plausible network models of the auditory processing hierarchy to whole-scalp event-related potential recordings. This allowed us to identify regionally specific effects of ketamine in a distributed network of interacting cortical sources.

**Results:**

We show that the effect of ketamine is best explained as a selective change in intrinsic inhibition, with a pronounced ketamine-induced reduction of inhibitory interneuron connectivity in frontal sources, compared with temporal sources. Simulations of these changes in an integrated microcircuit model shows that they are associated with a reduction in superficial pyramidal cell activity that can explain drug effects observed in the event-related potential.

**Conclusions:**

These results are consistent with findings from invasive recordings in animal models exposed to NMDAR blockers, and provide evidence that inhibitory interneuron–specific NMDAR dysfunction may be sufficient to explain electrophysiological abnormalities induced by NMDAR blockade in human subjects.

*N*-methyl-D-aspartate receptor (NMDAR) hypofunction is considered one of the primary causes of schizophrenia 1, 2, which itself is associated with a number of electrophysiological brain abnormalities 3, 4. NMDAR antagonism, e.g., with ketamine, can reproduce a set of symptoms and electrophysiological features of schizophrenia 5, 6, 7, including a reduction in auditory mismatch negativity (MMN) observed in patients 8, 9, 10, 11. MMNs are difference waves of event-related potentials (ERPs) to an unexpected deviant stimulus and repeated standard stimuli 12, 13. One theory of the underlying perceptual inference is formalized in the predictive coding framework 14, 15: based on Helmholtz’s notion that the brain attempts to infer the causes of sensations 16, 17, predictive coding proposes that the brain generates predictions of sensory input. When sensation deviates from these predictions, prediction error signals are generated and are passed along the sensory hierarchy. Evidence from different sensory domains and species suggests that this provides a good explanation of MMN-type responses 18, 19. However, there is limited evidence on how the underlying mechanisms are affected by NMDAR transmission and its blockade.

Computational models offer a bridge between putative synaptic disease mechanisms, and electrophysiological and psychopathology features of disease phenotypes 20, 21. Dynamic causal modeling is one such approach: neural mass models of cortical microcircuitry are fitted to ERP data 22, 23, an approach widely applied to auditory MMN paradigms 24, 25, including in patients with schizophrenia (26), patients with psychosis (11), and healthy volunteers exposed to ketamine (27).

Here, we apply novel dynamic causal modeling (DCM) techniques to a double-blind, placebo-controlled study of the effects of ketamine on the auditory MMN. We employ a single hierarchical model to identify 1) within-session coupling changes explaining ERPs to deviant and standard stimuli (modeling MMN and repetition suppression effects respectively) and 2) groupwise between-session coupling differences induced by ketamine. Previous DCM studies have focused on synaptic changes plausibly affected by sensory input directly. Our approach accommodates more fundamental effects of ketamine on neuronal circuits; e.g., on excitation/inhibition balance within cortical microcircuits, on intrinsic timescales of cortical areas, or on postsynaptic gain. We exploit the biophysically detailed microcircuit models implemented in DCM, linking neurobiological insights and theoretical accounts of sensory processing in the brain (28). Thus, we use DCM to identify intrinsic (within-source) or extrinsic (between-source) synaptic connection changes induced by oddball stimuli, and how these changes were contextualized by altered neurobiology during the administration of ketamine.

With this approach we addressed two main hypotheses. First, ketamine may alter synaptic connectivity of the auditory processing network irrespective of the auditory context. Because of the nonlinearities of neuronal systems, even a context-invariant effect on connectivity (i.e., in parameter space) may produce differential effects in standard versus deviant responses in terms of ERPs (i.e., in measurement space). Second, and alternatively, ketamine and the ensuing NMDAR blockade may directly impact on synaptic plasticity induced by sensory learning. In other words, observed changes in deviant ERPs may be an interaction between deviant effects and drug effects at the level of synaptic connectivity. Given that our DCM approach estimates both synaptic plasticity induced by deviant stimuli and changes induced by ketamine, we can now disambiguate these putative mechanisms empirically.

## Methods and Materials

### Subjects

We recruited 18 male volunteers (Table 1) through university advertisements. Subjects gave fully informed, written consent prior to participation and were compensated. The study was approved by the University of Lübeck Research Ethics Committee. Participants completed a psychiatric questionnaire (Symptom Checklist-90-Revised) and routine clinical examination (including electrocardiography, auscultation, and blood pressure measurements). Subjects with preexisting conditions, a family history of psychotic illness or epilepsy, and regular medication, and who were left handed, smoked, or were recreational drug users, were excluded. Participants were invited for two sessions >3 weeks apart (placebo and ketamine arms).

**Table 1:** Study Subject Details

ID	Dose (mg/kg/h)	Age (Years)	Sex
A	0.250	23	Male
C	0.250	21	Male
E	0.250	23	Male
G	0.083	22	Male
I	0.083	22	Male
J	0.250	22	Male
K	0.083	23	Male
M	0.083	26	Male
N	0.083	22	Male
O	0.083	24	Male
P	0.083	25	Male
R	0.083	22	Male
T	0.083	22	Male
U	0.083	22	Male
W	0.083	25	Male
X	0.250	21	Male
Y	0.250	25	Male
Z	0.083	23	Male

This was a randomized, placebo-controlled, double-blind, crossover experiment: Participants were randomly assigned to either ketamine-first or placebo-first groups (9 in each group). Subjects and supervising researchers were blinded to session conditions (N.B., psychotomimetic effects of ketamine may reveal session conditions to participants). Ketamine or the saline placebo was infused continuously over 2.5 hours, with ERP recording commencing at 1.5 hours after onset 29, 30. An initially used higher ketamine dose was poorly tolerated by some participants, who suffered from nausea, vomiting, and some degree of disorientation. Because of this, the concentration of the infusion was reduced from 0.250 to 0.083 mg/kg/hour for subsequent data collection (Table 1).

### Stimuli and ERP Recording

ERPs were recorded using 20 electrodes (10/20 EASYCAP system [EASYCAP GmbH, Herrsching, Germany]; Compumedics Neuroscan amplifier, sampling frequency 500 Hz [Compumedics Neuroscan, Abbotsford, Australia]). Pure tones were presented in pseudorandom sequences: sounds of the same frequency were repeated 2 to 36 times, before a frequency change (i.e., roving oddball paradigm). Sounds were presented at 80 dB, with frequencies between 700 and 1200 Hz (in 50-Hz steps), and an interstimulus interval of 400 ms; tones were 25 ms in duration (31). Subjects were given an incidental reading task and instructed to ignore the sounds.

Data were analyzed in average referential montage, bandpass filtered (range, 0.1–80 Hz), and divided into −100- to 300-ms peristimulus epochs. In the roving paradigm, tones change from deviant stimuli (i.e., first in sequence) to standard stimuli through increasing repetitions. We calculated average ERPs for the first (deviant [D1]; average of 228 trials per participant), second (S2), sixth (S6), and 36th (S36) (average of 76 [S36] to 209 [S2] trials per participant) presentation in a sequence. Baseline correction was performed based on 100- to 0-ms peristimulus time (D1), 250 to 300 ms (S2), or both (S6, S36) to avoid large P3a components at the end of D1 and the beginning of S2 epochs.

### Experimental Design and Statistical Analysis

The experiment was designed to determine within-session effects of stimulus repetition and deviance, as well as between-session effects of ketamine in a crossover design. Within sessions, we compared ERPs with standard tones after short (two tones) and long (36 tones) sequences, as well as ERPs after 36 tones, and the deviant ERP (first tone) using Bonferroni-corrected *t* tests for time point by time point differences. To evaluate between-session group effects of ketamine versus placebo, we compared peak amplitude of the mean difference between the standard and deviant ERPs (i.e., mismatch negativity) with a *t* test.

### Dynamic Causal Modeling

Further detailed analysis using cortical source estimates of population output was performed using DCM. Our analysis was based on low-density electroencephalogram (EEG) recordings (20 electrodes in the 10/20 electrodes system), resembling EEG recordings routinely used in the clinic. These data were sufficient for our purposes because the aim of our study was not to identify the functional architecture of the auditory mismatch response, which has been done before 32, 33. Rather, we wanted to infer the effects of ketamine on the neurobiology of an established MMN network. Our models reflect this question, focusing on variations in model parameters as the explanation for the ERP differences. Prior knowledge about source locations was included in the DCM inversion as before, enabling us to finesse the source reconstruction problem using low-density EEG data and thereby drill down on the ketamine effects. We apply hierarchical (parametric empirical) Bayesian modeling to identify group effects across DCMs fitted to single subjects. This analysis was conducted using the free academic software SPM12 (http://www.fil.ion.ucl.ac.uk/spm/), and custom code available online (https://doi.org/10.5281/zenodo.570595).

#### Identifying Prior Parameter Distributions From Grand Mean ERP

To produce the best fits at the single-subject level, a DCM was first fitted to grand mean ERPs 24, 33, as detailed below. A standard electromagnetic forward model based on a boundary element method standard head model in Montreal Neurological Institute space implemented in SPM12 was used to calculate lead-fields and reconstruct source ERP waveforms at six cortical locations 25, 34: bilateral primary auditory cortex (A1), bilateral superior temporal gyrus (STG), and bilateral inferior frontal gyrus (IFG) with Montreal Neurological Institute coordinates: left A1 [−42, −22, 7], right A1 [46, −14, 8], left STG [−61, −32, 8], right STG [59, −25, 8], left IFG [−46, 20, 8], right IFG [46, 20, 8].

**Figure 1:**
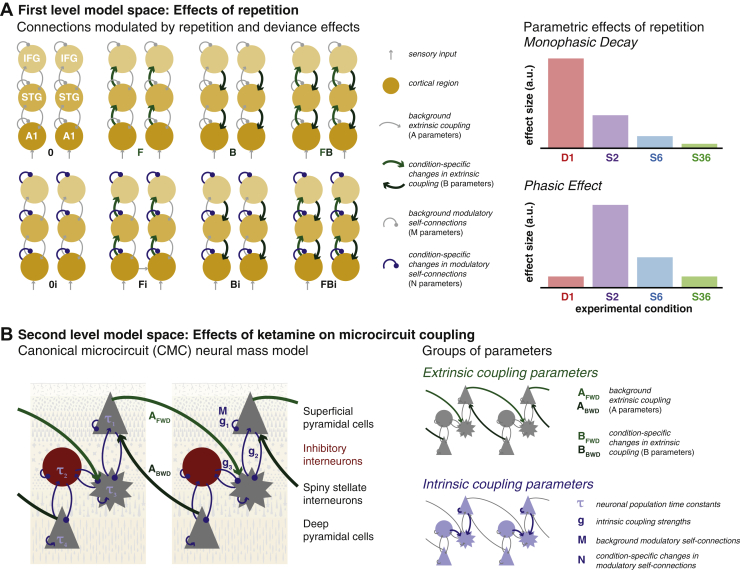
Two-level hierarchical dynamic causal modeling (DCM) model space. DCM represents cortical sensory processing as a network of (extrinsically) coupled cortical sources, each of which contains (intrinsically) coupled neuronal populations. Each population is modeled as a neural mass with characteristic synaptic time constants. The free parameters of this model include extrinsic coupling parameters between cortical sources (average coupling: A parameters; condition-specific modulation: B parameters) and intrinsic coupling parameters between neuronal populations within a source (population time constants: τ parameters; coupling strengths: g parameters; activity-dependent modulation of recurrent self-connections: M parameters; condition-specific modulation of activity-dependent coupling: N parameters). **(A)** Effects of repetition were modeled as condition-specific modulations of forward/backward extrinsic connections (B_BWD_/B_FWD_) (B parameters) and intrinsic modulatory gain parameters (N parameters) in a coupled network of six cortical sources. The relative contribution of these parameters to each of the event-related potentials modeled here (i.e., D1, S2, S6, S36) is estimated using two temporal basis functions: a monophasic decay (where the contribution is maximal for the deviant stimulus D1) and a phasic change in connectivity (where the response is maximal at the standard stimulus S2), as shown in the right panels. **(B)** Ketamine effects were modeled at the second level; i.e., as group-level differences in DCM parameters between conditions that were conserved over subjects. The left panel illustrates the parameters in the canonical microcircuit representation of each source used in this DCM. Our analysis addressed the following question: which combination of parameter changes between the placebo and ketamine conditions best explains the ketamine effect observed across the whole group? The model space is divided into models in which ketamine affects only combinations of extrinsic coupling parameters (right top panel) versus those in which ketamine affects only combinations of intrinsic coupling parameters (right bottom panel). a.u., arbitrary units; A1, primary auditory cortex; A_BWD_, extrinsic backward coupling; A_FWD_, extrinsic forward coupling; B, backward connections; F, forward connections; i, intrinsic connections; IFG, inferior frontal gyrus; STG, superior temporal gyrus.

Differences between ERPs were modeled as arising from extrinsic (between cortical regions) or intrinsic (within cortical regions) synaptic coupling change. We modeled the effect of deviance and repetition as coupling changes (i.e., short-term plasticity) that are summarized as linear mixture of two temporal basis functions (Figure 1A). This yields three types of coupling: subject-specific connectivity conserved across repetitions (A parameters), repetition-dependent changes in connectivity greatest for the deviant and subsequently decreasing (B parameters for the monophasic decay), and repetition-dependent changes in connectivity that peak with the first standard tone S2 (i.e., B parameters for the phasic temporal basis function). The linear mixture of both types of temporal basis function—weighted by their respective B parameters—reproduces the estimated connectivity changes across the four conditions (i.e., repetitions) allowing for a range of different types of plasticity over time (33). The model inversion provides both a measure for model evidence and posterior densities of model parameters. Parameter estimates of this grand mean inversion were then used as priors for single-subject DCMs.

#### Individual Model Inversion and Bayesian Model Reduction to Identify Repetition Effects

Whole-scalp ERPs for each subject were extracted separately for the placebo and the ketamine conditions, resulting in 36 separate sessions (18 subjects, two conditions) for DCM analysis. For each DCM, the full six-region network was equipped with grand mean–derived priors and inverted, yielding 36 individually parameterized DCMs.

To test whether the effects of deviance (24) and the effect of repetition (33) replicate existing findings in the literature, we performed Bayesian model reduction. This identifies the best subset of DCM parameter changes that could explain the observed ERP responses: based on inverted full DCMs, we can estimate model evidence for a number of reduced DCMs, in which some parameters do not allow condition-specific variations 23, 35, 36.

We compare models in which repetition modulated only a subset of network connections along three classes of models—1) models in which forward connections change versus models in which they do not, 2) models in which backward connections change versus models in which they do not, and 3) models in which intrinsic modulatory gain parameters change versus those in which they do not 24, 25. This furnishes a combination of 2 × 2 × 2 = 8 models: four types differing in between-source connectivity modulation and two types differing in within-source connectivity modulation (Figure 1A). As each subject had a high model evidence for the same (winning) model (see Results), within-session effects across the group were summarized using Bayesian parameter averages.

#### Parametric Empirical Bayes and Ketamine Effects

To estimate systematic variations in model parameters caused by ketamine, we used a parametric empirical Bayesian (PEB) approach. In brief, PEB allows the Bayesian estimation of a general linear model explaining effects across DCMs at the level of model parameters. This second-level model can be equipped with different regressors (across all sessions and subjects), with the inversion providing parameter estimates for these between-DCM effects (37). Here, we use PEB to 1) perform Bayesian model comparison across reduced models and 2) quantify the parameter changes in the winning model in which a subset of DCM parameters explains the ketamine effect.

Regressors comprised 1) an effect of ketamine (0 for placebo, 1 for low-dose ketamine, 2 for high-dose ketamine; the two sessions from each individual subject were thus modeled as either 0 to 1, or 0 to 2, thus preserving differential effect sizes of ketamine across participants), 2) the group mean, and 3) random subject or block effects. The parameters included time constants (τ_1–4_) (temporal dispersion of postsynaptic responses), intrinsic connectivity parameters (g_1–3_) (cortical microcircuit connection strengths), modulatory gain parameters (M, N) (modulations of superficial pyramidal cell gain), and extrinsic connectivity parameters (A, B) (connection strength between cortical sources). Our model space addressed two classes of hypotheses: 1) ketamine affects extrinsic connections between sources (A, B parameters) and 2) ketamine modulates intrinsic properties within sources (M, N, g, τ parameters). We allowed for both a nonspecific (main) effect of ketamine on coupling (M, A, g, τ parameters) and interaction effects specifically on plasticity (N, B parameters). We used Bayesian model reduction to compare the evidence for these (second-level) models, yielding approximate log evidence of second-level models and estimates of the ketamine-induced parameter changes (with Bayesian 95% confidence interval). Finally, to further characterize these effects, we used parameter estimates in simulation mode (i.e., in a forward model based on the grand mean DCM) to visualize their impact on source-space ERPs.

## Results

### Sensor Space Results

**Figure 2:**
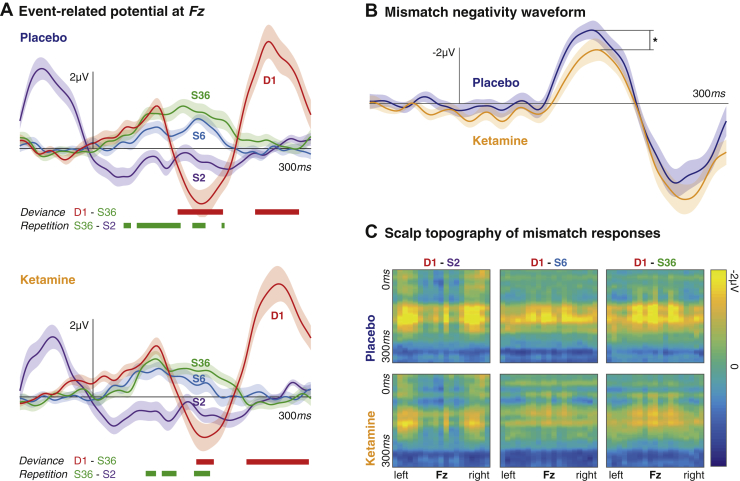
Ketamine causes a reduction in the mismatch negativity. **(A)** Event-related potentials are shown for repetitions of a sound within a roving oddball paradigm. The first exposure to a sound within a sequence (D1) provokes a typical deviance response at the Fz electrode. Event-related potentials for three different repetitions (S2, S6, and S36) show increasing positivity with a peak at approximately 120 ms. The bold red lines indicate time points for which the S36 and D1 event-related potentials are significantly different across the group (i.e., the effect of deviance; *p* < .05, Bonferroni corrected for multiple comparisons); the bold green lines indicate time points for which S36 and S2 are significantly different (i.e., the effect of repetition; *p* < .05, Bonferroni corrected for multiple comparisons, differences only tested for the 0- to 300-ms peristimulus time interval). Ketamine reduces both the deviance and repetition effects. **(B)** Difference waveforms at Fz are shown for D1 to S36. The peak amplitude of the waveform around 150 ms is significantly larger for the placebo condition compared with the ketamine condition (indicated by the asterisk). **(C)** The panels show the difference between D1 and S2, S6, and S36, respectively, across time (y-axis) and channels (x-axis, arranged from left to right). For each standard-deviant pair, there is a ketamine-related reduction in mismatch responses.

ERPs contain averaged responses to tones of different frequencies at fixed positions within a sequence. Grand mean ERPs at the frontocentral (Fz) electrode for D1, S2, S6, and S36 are shown for placebo and ketamine conditions (Figure 2A). Deviance ERPs (to D1) constitute an early negative response (at MMN latency, approximately 150 ms) and later positivity (P3a, approximately 250 ms) that differs significantly from ERPs to standard tones (deviance effect). ERPs to S2, S6, and S36 show the buildup of a positive memory trace at around 120 ms, with significant differences between S2 and S36 indicated in Figure 2A (repetition effect).

Ketamine reduced the period during which there was a significant deviance effect (i.e., D1 to S36 ERPs: placebo 112 ms, ketamine 90 ms) as well as the repetition effect (i.e., S26 to S2 ERPs: placebo 120 ms, ketamine 98 ms). The MMN is attenuated by ketamine (paired *t* test) (*t*_17_ = 1.85, *p* > .05) but not P3a (*t*_17_ = 1.10, *p* > .05) (Figure 2B). The attenuation is also apparent across the whole scalp when plotting all channels (Figure 2C).

### Effects of Repetition on Connectivity

**Figure 3:**
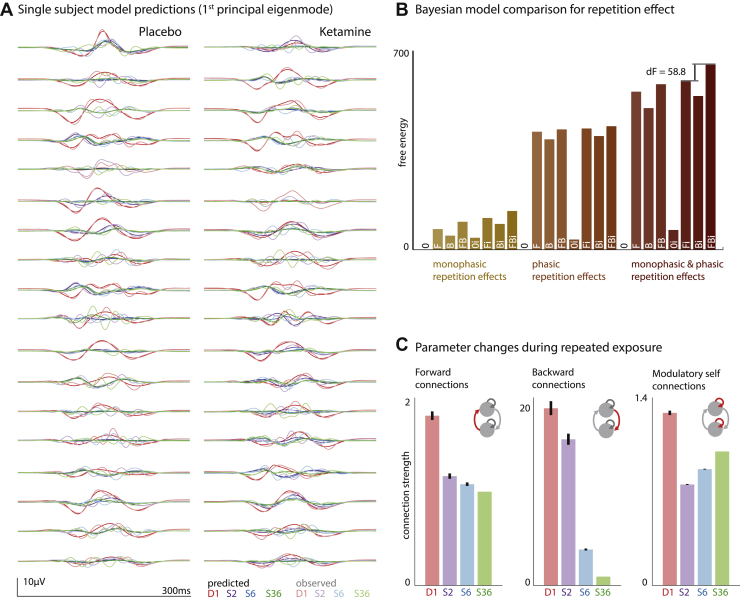
Repetition effects. **(A)** Event-related potentials to the first (deviant stimulus D1), second, sixth, and 36th (standard stimuli S2, S6, and S36, respectively) presentation of a sound within a sequence were modeled in subject-specific dynamic causal models. The first principal eigenmode of the prediction in sensor space (bold colors) and the corresponding mode of the empirical scalp data (light colors) are shown for each individual. These suggest a good fit for the main components of the event-related potential waves. **(B)** Bayesian model comparison was performed to compare models in which the repetition effect was monophasic, phasic, or both, and included modulations of forward (F), backward (B), or intrinsic (i) connections and their combinations. The winning model across the group was the full model, where monophasic and phasic repetition effects impact on forward, backward and intrinsic connection. **(C)** Bayesian parameter averages for this full model of all subjects show changes in connection strength across repetitions for forward, backward and intrinsic modulatory connections. Error bars indicate the 95% Bayesian confidence interval.

Repetition effects were modeled as changes in connectivity of the cortical auditory network comprising three bilateral sources. This plasticity is captured in a linear mixture of two temporal basis functions (Figure 1A): a monophasic decay and a phasic effect across repetitions. The combination of both effects on extrinsic and intrinsic coupling constitute the full model (model FBi in Figure 1A; see Figure 3A for subject-specific model fits). A set of reduced models was compared using Bayesian model reduction. These models comprised each of the models in Figure 1A paired with either one, or both, of the temporal basis functions, resulting in a total of 8 × 3 = 24 models. Bayesian model comparison provides decisive evidence for the full model (i.e., FBi with both monophasic and phasic effects) at the group level (Figure 1B) and for each individual subject (not shown).

Bayesian parameter averages for forward connections, backward connections, and modulatory self-connections (shown here for A1) are shown in Figure 3C, indicating distinct time courses of changes for different types of connections. Overall, extrinsic connectivity was reduced across repetitions: biggest reductions were seen earlier in forward compared with backward connections. Modulatory gain parameters are reduced initially (D1 to S2), before increasing (S2 to S36). The combination of these parameter best explains the observed ERP changes with repetition.

### Effects of Ketamine on Model Parameters

**Figure 4:**
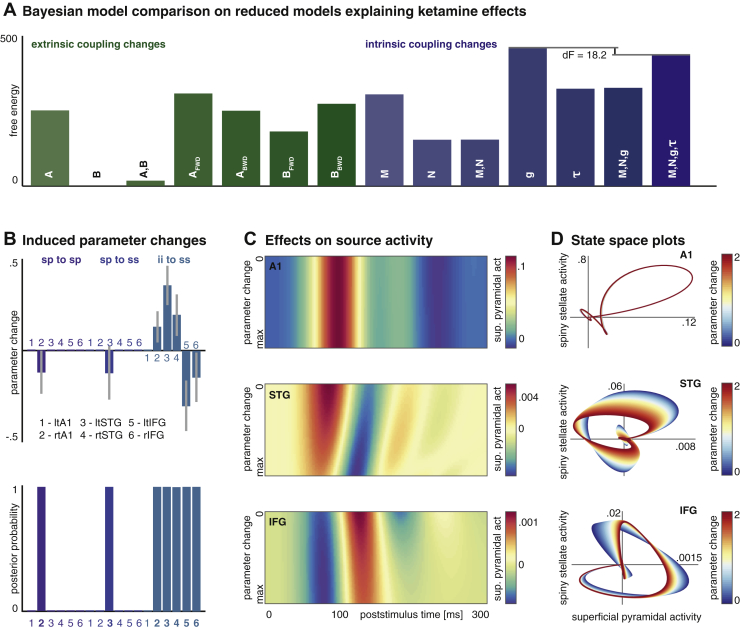
Ketamine causes frontal lobe disinhibition. **(A)** Using parametric empirical Bayesian modeling, 14 alternative second-level models were considered, explaining differences between ketamine and placebo with changes in combinations of parameters. Bayesian model reduction shows that the model with changes in intrinsic connection parameters (g) best explains the effects of ketamine on the event-related potentials. **(B)** Estimated parameter changes with Bayesian 95% confidence intervals (top) and posterior probability of the parameter being affected by ketamine (bottom) are shown. Significant changes were only observed in a subset of g parameters, with the largest effects estimated for inhibitory interneuron (ii) connections to spiny stellate cells (ss). In the bilateral superior temporal gyrus (STG), there was an increase in ii inhibition on ss, while in the bilateral inferior frontal gyrus (IFG) there is a ketamine-induced disinhibition of ss. **(C)** Simulated effects of opposing changes in ii to ss inhibition at different hierarchical levels are shown in source space. Each graph shows superficial pyramidal cell (sp) activity in different regions for the 0- to 300-ms poststimulus interval with concurrent, but opposite, modulation of the parameter in ii to ss inhibition: in the STG the (log-scaled) connection strength is increased from 0 to 2, while in the IFG the strength is decreased from 0 to −2. This modulation causes an attenuation and increase in latency in the IFG response, with concurrent attenuation of early STG responses and a decrease in the latency of the response. **(D)** Neuronal state space plots show the relationship between sp and ss activity for different hierarchical levels and for increasing changes to the ii to ss inhibition. There is minimal effect on the primary auditory cortex (A1). For STG, the parameter changes induce a reduction in ss response amplitude compared with sp and an overall shift toward more negative population output. In the IFG there is an inverse reduction of sp response amplitude compared with ss. τ, time constants; A_BWD_, extrinsic backward coupling; act, activity; A_FWD_, extrinsic forward coupling; B_BWD_, condition-specific modulations of backward extrinsic connections; B_FWD_, condition-specific modulations of forward extrinsic connections; lt, left; M, modulatory self-connections; N, condition-specific effects on modulatory connections; rt, right.

We combined DCMs for each subject and session in a single PEB model to identify between-session parameter changes induced by ketamine. Initially, we used Bayesian model reduction at this second level to compare reduced models that contained ketamine-related variation in only a subset of coupling parameters (Figure 4A). This allowed us to identify the simplest models with greatest explanatory power: in our hypothesis space we allow for complex interactions between ketamine and the effects of repetition suppression on the network (through the selection of model parameters). For example, the N parameters of our DCMs encode repetition-induced changes in cortical self-modulation. The second-level (PEB) model space included models in which the ketamine effect could be explained through changes in these N parameters; e.g., ketamine could attenuate changes in self-modulation during repeated exposure to the same sound. However, the parameters encoding these effects were redundant and were eliminated after Bayesian model reduction. A better explanation for the ketamine effects on the MMN was instead a repetition-invariant change in cortical microcircuitry encoded in the g parameters.

An inspection of the winning second-level model revealed that of these g parameters, only a subset is affected by ketamine (Figure 4B). The biggest effect size is seen in g_3_, which represents the strength of inhibition supplied by inhibitory interneurons to excitatory spiny stellate cells. This parameter is modulated in the opposite direction in the lower areas of the hierarchy (increased in right A1, left and right STG; decreased in left and right IFG).

To simulate the (highly nonlinear) effects of these parameter changes on observed ERPs, we implemented a forward model based on the grand mean DCM inversion. This simulation gradually increased g_3_ in the bilateral STG, while decreasing the same parameter in the bilateral IFG. The effects of these reciprocal changes on source-space ERPs are shown in Figure 4C. This analysis reveals an attenuation and small increase in latency in the IFG response that resembles the observed changes in the MMN response at the Fz electrode in Figure 2B. The responses at the STG level are overall reduced in amplitude, with a decrease in response latency (10).

Further analysis of the relationship between excitatory interneurons (here modeled as spiny stellate cells) and the superficial pyramidal cells is shown in Figure 4D. Plotted in terms of estimated neuronal responses, these graphs represent the evolution of population responses during the deviance ERPs in neuronal state space, starting from and returning to baseline. These plots show the relative impact of g_3_ parameter changes. In the IFG, where interneuron inhibition on spiny stellate cells is reduced, this plot reveals a decrease in superficial pyramidal cell amplitude with relative preservation of spiny stellate cell activity. Conversely, in the STG, where g_3_ is increased, the amplitude of spiny stellate cells is relatively decreased compared with superficial pyramidal cells.

## Discussion

We identified region-specific changes in cortical microcircuits induced by ketamine during an auditory oddball paradigm. Our approach provides a unifying hierarchical model explaining network-wide short-term sensory learning effects, mismatch effects, and the effects of NMDAR-blockade with ketamine. We focused on auditory ERPs—but mismatch responses are known to be attenuated by ketamine in other modalities (38). Furthermore, our results indicate that ketamine affects background (i.e., condition-invariant) cortical circuitry. Our findings may thus represent generic aspects of how predictive processing is affected by ketamine, rather than domain-specific features of decoding the auditory environment.

### Computational Modeling Links Whole Brain Observations With Synaptic Mechanisms

Mesoscale neuronal models can reproduce various normal 39, 40 and abnormal 41, 42 brain responses, explaining complex sets of observations, such as ERP data (21). We used this approach to compare a range of possible ketamine effects on auditory ERPs.

Our results suggest that 1) acute NMDAR blockade effects can be explained by a small set of key parameters and 2) the parameters identified are consistent with a wealth of previous related, but methodologically distinct, studies. The nontrivial link between MMN changes and intrinsic inhibition within frontal microcircuits could not have been made without explicit computational modeling of EEG generators. With this approach, we provide evidence for a localized and cell type–specific role of NMDAR hypofunction in psychotic illnesses such as schizophrenia 43, 44 in a placebo-controlled human experiment.

### Deviance Responses Are Caused by Network-wide Connectivity Changes

Competing theories regarding the origin of the MMN can be summarized as 1) the neural adaptation hypothesis, according to which the MMN is explained by bottom-up dishabituation (45); and 2) the model adjustment hypothesis, according to which MMNs represent an error detection signal prompting predictive model updating (46). Here we replicate findings from previous studies pertaining to the neurobiological implementation of these putative mechanisms. In this study, we assume the same cortical sources previously identified and replicated in a number of auditory oddball EEG and magnetoencephalography studies 24, 25, 47, 48. This allowed us to focus on processing dynamics rather than network topology. Our placebo results support previous DCM studies of the MMN showing that both neural adaptation and model adjustment are required to explain the phenomenon, as it requires changes in both intrinsic modulatory gain and extrinsic cortical coupling. This is in keeping with a predictive coding account of MMN generation. According to the predictive coding framework, sensory predictions are passed downstream along the sensory processing hierarchy. When they mismatch sensory evidence, a prediction error signal is evoked, which passes back up the cortical hierarchy (modulating extrinsic connectivity), while also causing adjustment of the intrinsic gain in primary sensory cortex (modulating intrinsic connectivity) 17, 18.

### Sensory Learning Causes Distinct Patterns of Change for Different Coupling Parameters

In the roving oddball paradigm, deviant sounds are repeated until they become the new standard, no longer eliciting deviance responses. Previous DCM studies identified distinct temporal patterns in associated coupling changes: using short auditory sequences, Garrido *et al.* (33) identified a clear difference in extrinsic connections, which were consistently reduced with each repetition; and intrinsic connections, which showed an initial phasic decrease before slowly increasing with repetition.

These findings are replicated independently here: extrinsic connectivity decreases with repetition, while intrinsic connectivity parameters show only a brief phasic decrease. There is also a temporal dissociation between forward and backward connections: while forward connection strengths quickly return to their baseline value, backward connection strengths remain higher for longer. This asymmetry in the time course of forward and backward plasticity may reflect more general differences in temporal dynamics at different points along the cortical hierarchy. Primate cortical areas are hierarchically ordered in their neuronal time scales 49, 50. The resultant hierarchically segregated tracking of fast and slow changes at different levels of the hierarchy may support efficient representation of complex sensory input (51). Our findings add further support for this hierarchical separation in time scales. After the ERP to the deviant stimulus, short transient increases of forward connections reflect novel sensory information (encoded in prediction errors) in lower cortical areas. More persistent changes in backward connections, in contrast, encode the preceding sensory context (i.e., the recent occurrence of the deviant), which persists for number of repetitions.

### NMDAR Blockade Has Regionally Specific Effects on Intrinsic Connectivity

NMDARs are prevalent in the supragranular cortical layers, suggesting particular relevance of NMDAR transmission to backward connections in the cortical hierarchy, as they target superficial layers (52). However, NMDARs are unevenly distributed across cortical interneuron subtypes, indicating that the overall effects of systemic NMDAR blockade may be better represented in regionally specific intrinsic coupling changes (excitatory or inhibitory), corresponding to localized subpopulation effects, rather than extrinsic coupling (53).

In our study, changes in a limited set of regional intrinsic connections best explain the ketamine effects, with strongest effects in a single connection type: inhibitory connections from inhibitory interneurons to spiny stellate interneurons. This is one of the links between fast-oscillating superficial and slow-oscillating deep neuronal oscillators of cortical microcircuits (54). This effect is region specific: ketamine causes a decrease in inhibitory interneuron to spiny stellate inhibition in the STG, but an increase in the IFG, indicating a relative disinhibition of the IFG. Our findings contain within them a replication of the work by Schmidt *et al.* (27), who considered a subset of model parameters for the ketamine effect. They identified a single forward connection as the ketamine-induced change in connectivity during a roving oddball paradigm. However, in our more comprehensive model space, the regionally distinctive disinhibition proves a more parsimonious explanation of ketamine effects. This is furthermore in keeping with previous DCM studies of ketamine in other model organisms also implicating prefrontal regions 55, 56, as well as an MMN study in people with psychosis and their relatives (11).

Functionally, the ketamine-related reduction in prefrontal inhibition results in a constitutive increase in prefrontal excitability, or gain. Dysfunctions in gain control have been put forward as explanations for aberrant sensory processing underlying hallucinations (57), explaining hallucinations as a failure to encode sensory uncertainty. Several clinical features of psychosis, including attenuated mismatch negativity, can be explained through hierarchical failures in precision encoding, as discussed in detail in Adams *et al.* (58).

Interestingly, cell type–specific knockouts of NMDAR on inhibitory interneurons are already used in animal models of schizophrenia 7, 59. Invasive recordings in the prefrosdntal cortex of such mouse models suggest that the overall effect of NMDAR transmission is an inhibitory drive (60). Detailed examination of the role of NMDAR blockade on gamma oscillations (known to be abnormal in schizophrenia) also showed the effect to be mediated through inhibitory interneurons (61). Although—by design—our study focused on evoked and not oscillatory responses, we note the convergence on inhibitory dysfunction in these optogenetic mouse models, and EEG studies in human subjects [e.g., 10, 60, 61]. The regional specificity of our effects at the source level is furthermore mirrored by a literature on topographically specific impairments in schizophrenia 62, 63. Prefrontal inhibitory interneuron dysfunction has also emerged as a potential mechanism underlying other features of schizophrenia 64, 65, further supported by computational models of prefrontal cortex functions (66).

The focus of this study was to relate pharmacological perturbation of brain function with measurable ERP modulations. Our results concerning the localizable effects of NMDAR blockade have clear implications for NMDAR-focused hypotheses of schizophrenia. While previous studies have shown a link between MMN measures of abnormal physiology in schizophrenia and psychopathology (67), we have not specifically addressed psychopathology in the test subjects here. Previous studies have shown that MMN features (68) or MMN-derived DCM parameters (27) during ketamine exposure correlate with different aspects of psychopathology, which is clearly an important area for future research.

### Limitations

This study uses complex DCMs on low-density EEG, limiting robust source localization without prior assumptions. Here, we are assuming cortical sources to be located at previously identified Montreal Neurological Institute coordinates 25, 34, 47, 48, focusing on exploring processing dynamics rather than MMN topology.

First, Bayesian model selection—as used in this study—can only provide relative evidence of models in the model space. A more parsimonious explanation of the data may exist, but we cannot comment on alternative hypotheses that are not included explicitly. We chose our model space carefully, based on the MMN literature to 1) accommodate previous findings on repetition suppression and the deviance response and 2) test specific hypotheses about the effects of ketamine in this setting. In short, we cannot draw conclusions about whether other models may offer better explanations for our data; however, we can argue that the model space offers a broad repertoire that includes most neurobiologically plausible hypotheses currently entertained in the literature.

Second, because of side effects for some of our participants, the dose of ketamine had to be adjusted for subsequent subjects. This additional variation is explicitly accommodated in the model (as a specific parameter in the parametric empirical Bayesian analysis), thus allowing integration of two distinct ketamine doses compared with placebo. Furthermore, drug-level monitoring was not included in the study design, so some intersubject variability may be accounted for by differences in drug metabolism and excretion. However, the statistical modeling used in our analysis accommodates the ensuing random (between-subject) effects.
